# Dapagliflozin added to metformin reduces perirenal fat layer in type 2 diabetic patients with obesity

**DOI:** 10.1038/s41598-024-61590-6

**Published:** 2024-05-11

**Authors:** Guillem Cuatrecasas, Francisco De Cabo, M. José Coves, Ioana Patrascioiu, Gerardo Aguilar, Gabriel Cuatrecasas, Sonia March, Marta Calbo, Olga Rossell, Mariona Balfegó, Camila Benito, Silvana Di Gregorio, Pilar Garcia Lorda, Elena Muñoz

**Affiliations:** 1 CP Endocrinologia SLP, 08037 Barcelona, Spain; 2 Center for Obesity Management EASO, Clinica Sagrada Familia, Barcelona, Spain; 3 Facultat Ciencies Salut, Open University Catalonia (UOC), Barcelona, Spain; 4 Ultrasound Department, Institut Guirado for Radiology, Barcelona, Spain; 5 EAP Sarrià, Barcelona, Spain; 6 Cognitive NeuroLab, Barcelona, Spain

**Keywords:** Ultrasound, Dapaglifozin, Metformin, Preperitoneal fat, Omental fat, Perirenal fat, Metabolic syndrome, Cardiovascular risk, Diabetes, Metabolic syndrome, Obesity, Ultrasound

## Abstract

Sodium-glucose co-transporters type 2 inhibitors (SLGT2i) are highly effective in controlling type 2 diabetes, but reported beneficial cardiovascular effects suggest broader actions on insulin resistance. Weight loss may be initially explained by glycosuria-induced net caloric output and secondary volumetric reduction, but its maintenance could be due to loss of visceral fat mass. Structured ultrasound (US) imaging of abdominal adipose tissue (“eco-obesity”) is a recently described methodology used to measure 5 consecutive layers of abdominal fat, not assessable by DEXA or CT scan: superficial subcutaneous (SS), deep subcutaneous (DS), preperitoneal (PP), omental (Om) and right perirenal (RK). PP, Om and RK are predictors of metabolic syndrome (MS) with defined cut-off points. To assess the effect of SLGT2i on every fat depot we enrolled 29 patients with type 2 Diabetes (HbA1c 6.5–9%) and Obesity (IMC > 30 kg/m^2^) in an open-label, randomized, phase IV trial (EudraCT: 2019-000979-16): the Omendapa trial. Diabetes was diagnosed < 12 months before randomization and all patients were treatment naïve. 14 patients were treated with metformin alone (cohort A) and 15 were treated with metformin + dapaglifozin (cohort B). Anthropometric measures and laboratory tests for glucose, lipid profile, insulin, HOMA, leptin, ultrasensitive-CRP and microalbuminuria (MAL) were done at baseline, 3rd and 6th months. At 6th month, weight loss was −5.5 ± 5.2 kg (5.7% from initial weight) in cohort A and −8.4 ± 4.4 kg (8.6%) in cohort B. Abdominal circumference showed a −2.7 ± 3.1 cm and −5.4 ± 2.5 cm reduction, respectively (*p* = 0.011). Both Metformin alone (−19.4 ± 20.1 mm; −21.7%) or combined with Dapaglifozin (−20.5 ± 19.4 mm; −21.8%) induced significant Om fat reduction. 13.3% of cohort A patients and 21.4% of cohort’s B reached Om thickness below the cut-off for MS criteria. RK fat loss was significantly greater in cohort B group compared to cohort A, at both kidneys. Only in the Met + Dapa group, we observed correlations between Om fat with leptin/CRP/MAL and RK fat with HOMA-IR. US is a useful clinical tool to assess ectopic fat depots. Both Metformin and Dapaglifozin induce fat loss in layers involved with MS but combined treatment is particularly effective in perirenal fat layer reduction. Perirenal fat should be considered as a potential target for cardiovascular dapaglifozin beneficial effects.

## Introduction

Sodium-glucose co-transporters type 2 inhibitors (SLGT2i) are highly effective in controlling type 2 diabetes, mainly by inducing net glucose output at the proximal tubule^1^, but reported cardiovascular beneficial effects^2,3^ also suggest broader mechanisms resulting in insulin resistance improvement^4^. Whether this is because of weight loss or pathways that involve sustained ketosis^5^, hyperglucagonemia^6^, hepatic signaling through fibroblast growth factor production^7^ or secretion of gastrointestinal incretin hormones^8^ still remains uncertain. We are talking about a new class of drugs with pleiotropic and additive actions in the control of body weight, glycemic levels, blood pressure and cardiovascular events^9^.

Weight loss may be initially explained by net caloric output produced with glycosuria and secondary volumetric excretion, but its maintenance is mainly due to loss of visceral fat mass^10^.

Ultrasonography (US) is an affordable technique that can be performed at the bedside of the patient, with an acceptable reproducibility, and is becoming a widely used method of anthropometry analysis in patients with Obesity^11–13^. It allows us to identify and measure 5 different stratified layers of abdominal fat (superficial subcutaneous (SS), deep subcutaneous (SP), preperitoneal (PP), omental (Om) and right perirenal (RK), not assessable by DEXA or CT scan, considered the gold-standard imaging tests but only able to separate *total* subcutaneous from *total* visceral fat. Do these separate anatomical fat depots explain different adipocyte functionality?^14^. Is their modification clinically relevant?

Immediately below dermis, superficial subcutaneous fat depot act as a protective barrier against infections, whereas deep subcutaneous, highly vascularized and innervated, is considered the major adiponectin-secreting fat depot^15^. It is also the location where higher number of preadipocytes can be found^16^ and where browning of adipose tissue takes place^17^.

**Figure 1:**
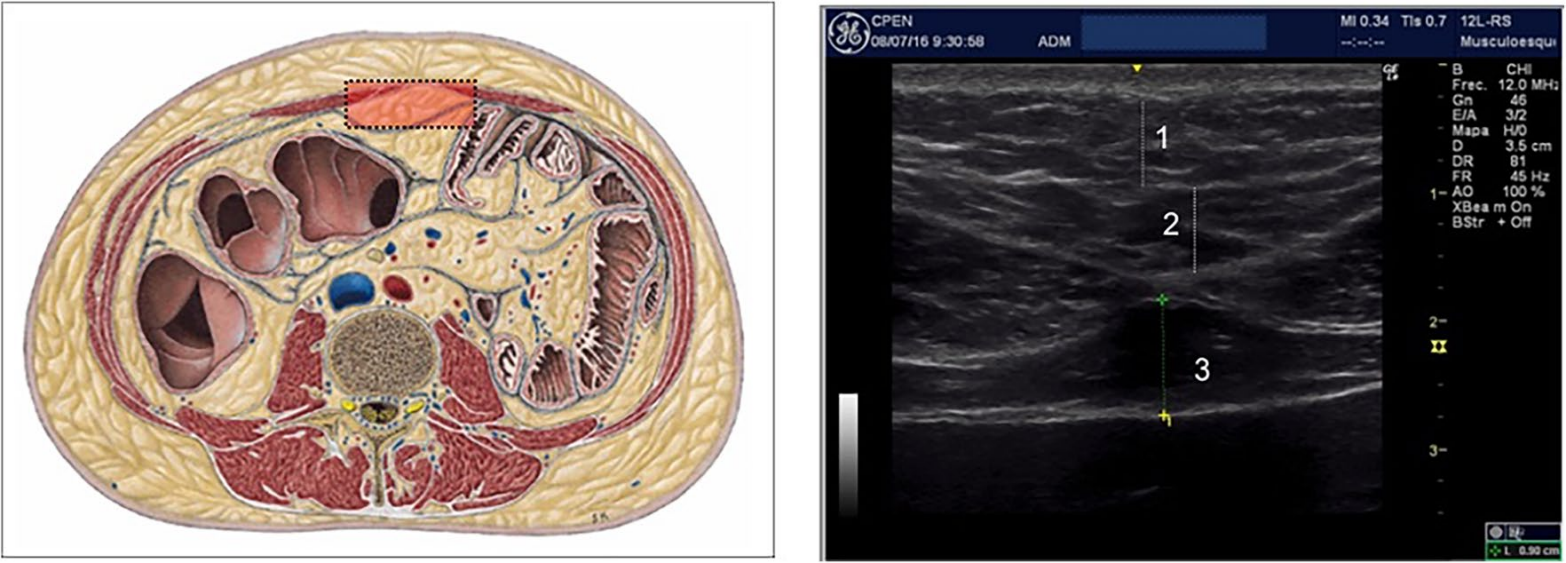
Subcutaneous and preperitoneal fat. 1.-Superficial subcutaneous fat layer. 2.-Deep subcutaneous fat layer. 3.-Preperitoneal fat layer.

The 3rd consecutive layer from the skin, Pre-peritoneal fat, markedly hypoechoic, has been related with endometrial cancer risk^18^, fatty liver disease^19^ and cardiovascular risk both in adults^20^ and adolescents^21^. Previously published cut-off limits of this fat depot for Metabolic syndrome at risk are 11 mm (men) and 9 mm (women)^11^ (Fig. 1).

**Figure 2:**
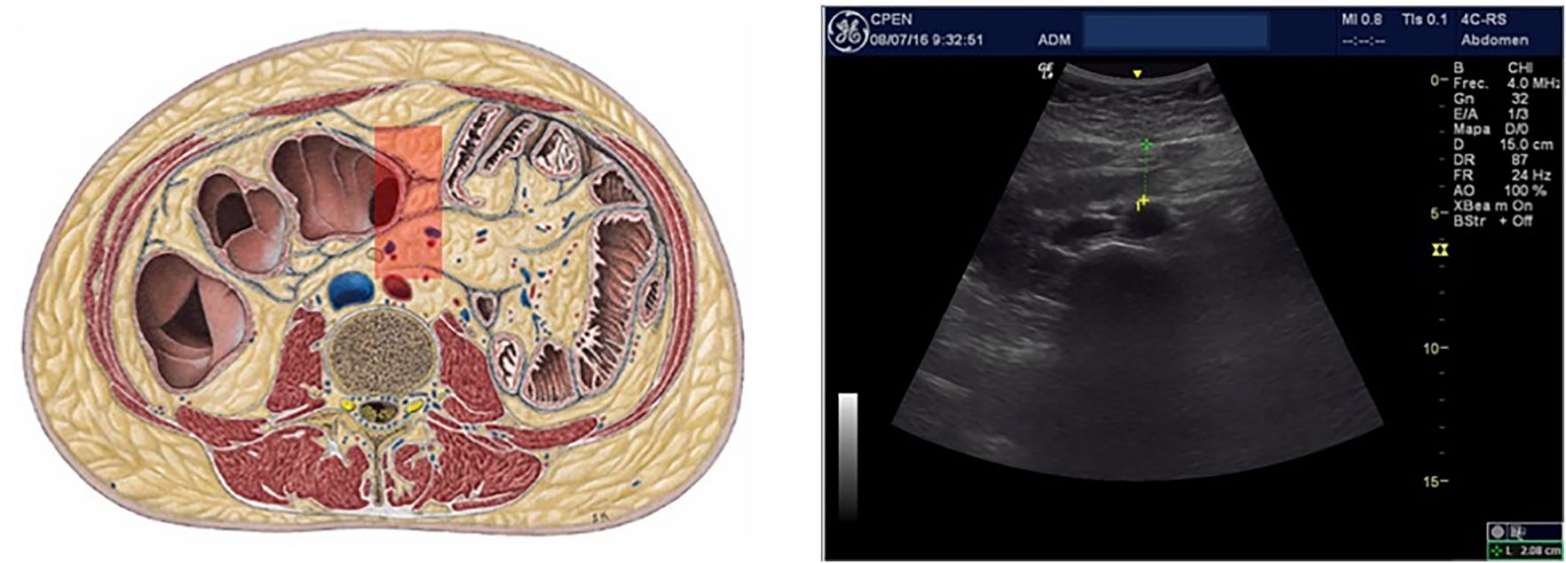
Omental (periaortic) fat.

Deeper inside the abdominal cavity, intra-peritoneal fat should be understood as 2 coexisting, melted, fat tissues: Mesenteric fat, rich in lymphoid and connective tissue that protects and fix abdominal organs to the dorso-lumbar posterior wall^22^ and Omental fat, metabolically active, surrounding bowels. This intraperitoneal fat is usually (but wrongly) called “visceral fat”, concept that should only refer to the fatty infiltration of solid organs such as liver, pancreas or kidney. It is known that Omental fat interacts with upper gastrointestinal tract and its microbiota, inducing insulin-resistance^23^ and is related with increased cardiovascular risk^24^. A specific cut-off measure for Omental fat thickness of 37 mm (women) and 54 mm (men), for Metabolic syndrome risk, has been proposed using ROC curves analysis^11^ (Fig. 2).

**Figure 3:**
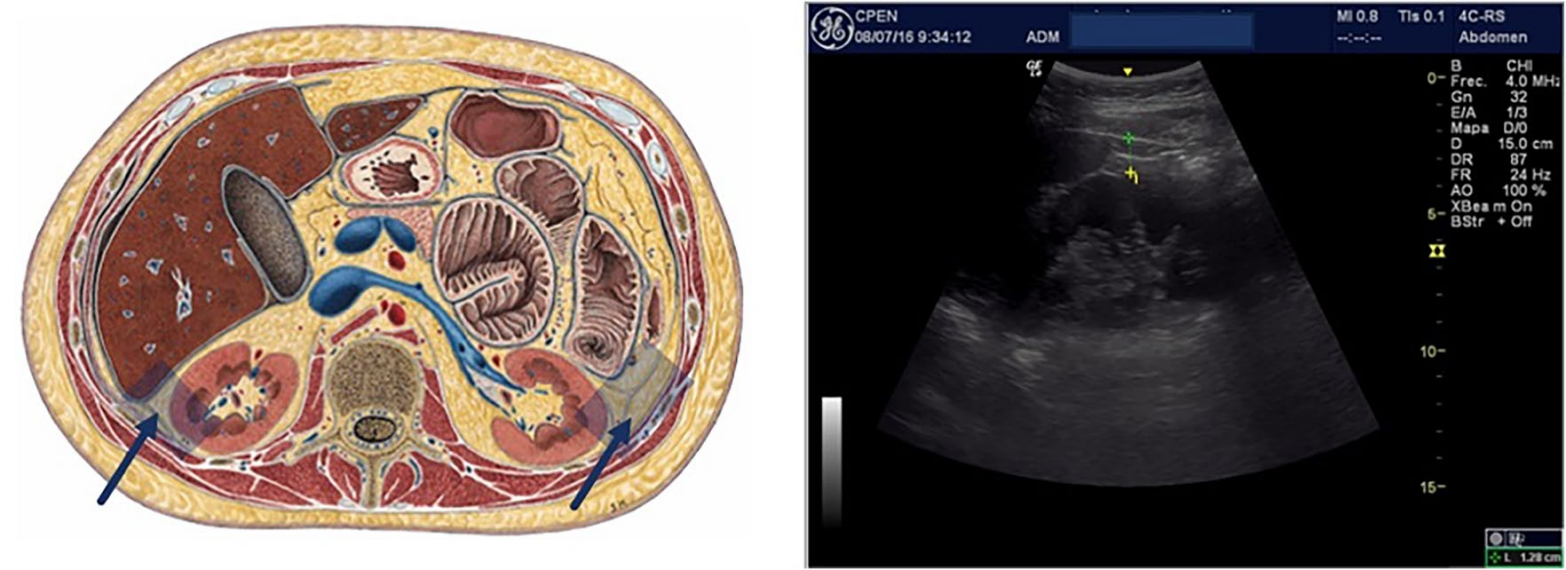
Perirenal fat.

Finally, in the retroperitoneal space, peri and para-renal fat (spliced by a fascia) surround both kidneys^25^. Perirenal fat thickness has also been related to Metabolic syndrome and cardiovascular risk^11^ with a proposed cut-off point of 17 mm (women) and 22.5 mm (men) (Fig. 3).

Cardiovascular risk association can be explained not only by differential adipokine expression but also by a mechanistic effect. A bigger perirenal fat depot may difficult a congestive kidney to expand, leading to increased intra-medullar pressure and consequent hypertension^26^.

Preliminary data of GLP1 receptor agonists treatment in patients with Obesity show a significant reduction of Preperitoneal, Omental and Perirenal fat thickness after 6th months of treatment, particularly in hypertensive patients^27^. GLP1a seems to act, not only reducing food intake but also as a major insulin-sensitizing agent^28^.

Nothing is known however about the specific effect of metformin or SLGT2i, alone and combined, on every layer of intra-abdominal fat. Both drugs have mild effect in weight loss but are first-line pharmacological options in Type 2 insulin-resistant diabetic patient treatment^29,30^. Can a differential action of Dapaglifozin in Preperitoneal, Omental or Perirenal fat explain cardiovascular improvement in combination-treated patients?

## Material and methods

*Omendapa* (EudraCT: 2019-000979-16) is a phase IV open-label randomized clinical trial, single-center study, to investigate the effect of some common antidiabetic drugs (Metformin and Dapagliflozin) in the reduction of the abdominal fat layers. According to study design, two different cohorts of type-2 Diabetes patients, drug-naive (only diet and exercise as current treatments) were analyzed. Patients were randomized for treatment initiation with Metformin alone (cohort “A”) at a maximum dosage of 1700 mg/day P.O (*n* = 14) or Metformin + Dapagliflozin 10 mg (cohort “B”) *n* = 15. All patients had a weekly dietary record to assess diet compliance and were prescribed the same hypocaloric diet with a −500 kcal/day deficiency. Dosage reduction of metformin to 850 mg/day was allowed in case of gastrointestinal side-effects.

Primary objective was to evaluate the possible differences between both arms of treatment (Metformin vs. Metformin + Dapagliflozin) regarding abdominal fat layers reduction from baseline to 6 months of treatment.

Secondary objectives were:

- Study correlation between fat depots thickness and cardiovascular risk measurements (biochemical risk markers and intima media thickness (IMT)) in both arms of treatment
- % patients who were able to reduce their omental fat below defined cut-off points ≥ 37 mm (females) and ≥ 54 mm (males) after 6 months of treatment
- Identify potential layers of abdominal fat that might correlate with glycemic control, clinical criteria for metabolic syndrome^31^, IMT or biochemical cardiovascular risk markers.

Inclusion criteria were:

- Patients of both sexes between the ages of 50 and 75 (both included) at the time of signature of informed consent (IC) form.
- Diagnosed of type-2 diabetes (defined as HbA1c level between 6.5 and 9%) a maximum of 12 months prior to IC form signature (median time was 9 month).
- Pharmacological treatment-naive patients (only diet and exercise as current treatments for glycemic control were permitted).
- BMI > 30 kg/m2 at baseline visit.
- Patients with omental fat > 37 mm (females) and > 54 mm (males) at baseline visit (pathological cut-off).

Exclusion Criteria were defined as follows:

Serious illness that according to investigator criteria could compromise the patient follow-up during the study.

Patient unable to follow the hypocaloric diet with a −500 kcal/day deficiency.

Previous use of other antidiabetic drugs.

- Hepatic failure defined as: Aspartate aminotransferase (AST), Alanine transaminase (ALT) or Bilirubin > 3X upper limits level compared to reference levels.
- Renal failure (defined as glomerular filtration (GF) < 60 ml/min).
- Concomitant use of other diuretic drugs.
- Recurrent Urinary Tract Infection (UTI) (defined as more than 2 UTI´s in last year previous to inclusion).
- Women of childbearing potential not practicing a highly effective method of birth control (A woman of childbearing potential must have a negative urine pregnancy test at screening within 10–14 days and 24 h before treatment initiation.

The study protocol was approved by the local ethics committee.

### Clinical data

*N* = 29 patients met all the selection criteria and were considered eligible to take part in the study. 51.7% were men and 48.3% women. Mean age was 61.2 ± 9.9 years (Table 1).

**Table 1:** Baseline Results.

Mean (Standard Deviation)	Cohort A (Met) *n* = 14	Cohort B (Met + Dapa) *n* = 15	*p*
Age	61.6 (9.3)	60.7 (10.7)	0.81
BMI	34.2 (4.9)	34.4 (4.7)	0.66
Abdominal circumference	112.7 (13.7)	113.0 (14.2)	0.96
% Body fat	49.1 (6.8)	47.9 (6.4)	0.96
Total Cholesterol (mg/dl)	183.3 (31.7)	219.8 (28.7)	0.003*
HDL (mg/dl)	48.3 (15.5)	48.2 (13.5)	0.99
Glucose (mg/dl)	139.6 (29.5)	142.2 (56.6)	0.88
HbA1c (%)	7.0 (1.2)	7.2 (0.9)	0.49
Insulinemia (uU/ml)	18.4 (11.0)	27.4 (10.2)	0.19
HOMA-IR	5.9 (3.3)	10.7 (6.2)	0.14
Leptin (ng/ml)	40.9 (37.6)	27.9 (18.2)	0.54
Ultra-sensitive CRP (mg/L)	4.5 (3.3)	4.4 (2.5)	0.93
Microalbuminuria (mg/24 h)	27.6 (45.3)	37.2 (96.9)	0.22
Superficial subcutaneous fat (mm)	15.4 (18.0)	10.0 (4.3)	0.61
Deep subcutaneous fat (mm)	18.0 (19.4)	19.8 (22.3)	0.51
Pre-peritoneal fat (mm)	4.1 (1.7)	5.1 (2.1)	0.22
Omental fat (mm)	85.1 (26.2)	86.0 (22.8)	0.92
Right Peri-renal fat (mm)	27.4 (8.9)	26.5 (9.1)	0.79
Right IMT (mm)	0.9 (0.2)	0.8 (0.3)	0.23
Left IMT (mm)	0.7 (0.2)	0.9 (0.3)	0.31

Abbreviations: BMI (Body mass index), IMT (intima media thickness), CRP (C-reactive protein).

### Anthropometric data

BMI was measured as kg/m^2^. Measures of mean abdominal perimeter (measured in cm, from top of both iliac crest) > 88 cm in female and > 102 cm in male were considered as major criteria for Metabolic syndrome definition^31^.

Total body fat was measured by multipolar bioelectrical impedanciometry (Inbody 530©), using 15 different measures (30 s. assessment) at 3 different frequencies (5–50–500 kHz) in left arm, right arm, trunk, left leg and right leg.

### Laboratory measurements

Measurements of basal glucose, HbA1c, total cholesterol, HDL and LDL-Cholesterol, Triglycerides, serum insulin, ultrasensitive-CRP, Leptin and Microalbuminuria were recorded at baseline and 6^th^ month, using ALINITY C reagents (ABBOT DIAGNOSTICS). Sensibility ranges were plasma insulin 3 uU/ml, ultrasensitive-CRP 0,1 mg/dl, Leptin 0.50 ng/ml and Microalbuminuria 3 mg/100 ml). HOMA index was also calculated and HOMA-IR > 4.05 (percentile 90) was considered as marker of insulin-resistance^32^.

### Ultrasound measurements

A General Electric Logic E© with a high frequency 12 MHz linear probe (superficial fat measures) and a 5 MHz Convex probe (intraperitoneal and retroperitoneal fat measures), was used for the abdominal fat ultrasound assessment. Following a strict standardized protocol, thickness (mm) of (a) total Subcutaneous tissue, divided into *superficial* and *profound subcutaneous* layers by the *fascia superficialis*^6^, (b) *Preperitoneal* fat, measured from the *linea alba* to the parietal layer of the peritoneum (Fig. 1), (c) *visceral* fat, including both *omental* and *mesenteric* fat and ranging from the peritoneal line to the anterior wall of the abdominal aorta (Fig. 2), and (d) *perirenal* fat, starting from the renal cortex to the triangle formed by liver pole and abdominal wall musculature (for the right *perirenal* depot) (Fig. 3), were sequentially measured^11^. All these measures were made where the abdominal aorta ultrasound imaging bifurcates into the iliac arteries, position corresponding to the 4th lumbar vertebra level (L4) using the usual reference point when measuring visceral fat with CT and MRI^33^. The correlation coefficient of the mean ultrasound distance assessed by two different sonographers at baseline is 0.94 (*P* < 0.001), with a mean difference 0.40 cm (SD 0.90), and a coefficient of variation of 5.40%, indicating good reproducibility of the ultrasound abdominal measurements^13^.

IMT was measured with the linear probe (12 MHz) at common carotid posterior wall (abnormal value: ≥ 0.9 mm) at both sides.

### Statistical analysis

The statistical study was carried out using the statistical software package SPSS version 19^34^.

All randomized patients who receive at least one dose of any study treatment (Metformin or Metformin + Dapagliflozin) have been included in the efficacy and safety analysis (Intention to treat). To analyze the statistical significance, change in the efficacy variables, different statistical methods have been applied according to the nature of the data. The comparison between groups of quantitative variables have been made using parametric (Student's t-test) or non-parametric tests (e.g., Mann–Whitney), based on the characteristics of the study variables (normality). For categorical variables, the chi-squared test or Fisher’s exact test have been applied. Pearson or Spearman correlation coefficient have been used to study the relation between quantitative variables. For comparisons between visits (baseline vs follow-up visits), parametric (paired Student’s t-test) or non-parametric test (Wilcoxon) have been used, based also on the characteristics of the variables (normality). All the hypothesis tests used for the efficacy endpoints have been performed considering 2-tailed tests and a significance level of 0.05.

### Ethical approval

This work was approved by the Ethical committee of Hospital Quiron-Teknon, Barcelona and approval was granted from the AEMP (Spanish regulatory agency). EudraCT: 2019-000979-16. AdKnoma® was the CRO that made the study follow-up according to Helsinki declaration and Good clinical practice guidelines. Informed consent was obtained from all subjects.

## Results

### Expressed as mean ± standard deviation

Mean BMI was 34.3 ± 4.7 kg/m^2^ and mean abdominal perimeter was 112.8 ± 13.6 cm at baseline. Baseline total body fat was 48.5 ± 7.8% (Table 1). Both groups had similar baseline abdominal fat layers thickness and were comparable at for lab measurements except for total cholesterol. All patients in both groups fulfilled the metabolic syndrome criteria (ATPIII). Hypertension was present at 57% of Group A and 61% of Cohort B. Total cholesterol was higher in Met + Dapa group, whereas HDL values were comparable (Table 1).

### Efficacy

At 6th month, weight loss was −5.5 ± 5.2 kg (5.7% from initial weight) in cohort A and −8.4 ± 4.4 kg (8.6%) in cohort B. Impedanciometry % fat mass reduction was −6.8 ± 4.8% and −10.4 ± 6.8 from baseline, respectively. No significant differences were seen comparing both cohorts.

Abdominal circumference showed a −2.7 ± 3.1 cm (cohort A) and −7.4 ± 2.5 cm (cohort B) reduction, with significant differences comparing the 2 groups (*p* = 0.011).

Both Metformin (−19.4 ± 20.1 mm; −21.7%) alone or combined with Dapaglifozin (−20.5 ± 19.4 mm; −21.8%) induce significant Omental fat reduction within groups (*p* < 0.05) but no differences were seen comparing the 2 cohorts.

21.4% of cohort’s A patients and 13.3% of cohort’s B reached Omental fat thickness below the cut-off for metabolic syndrome criteria (37 mm (females) and 54 mm (males)).

17.1% of cohort A patients and 27.3% of cohort’s B reduced perirenal fat below Metabolic syndrome cut-off point of 17 mm (women) and 22.5 mm (men).

Perirenal fat relative change (−20.4 ± 14.8% at left and −23.9 ± 9.2% at right kidney) was significantly higher in cohort B group compared to cohort A (−4.4 ± 21.0% at left and −8.5 ± 18.5% at right kidney) at both sides (*p* < 0.05).

**Figure 4:**
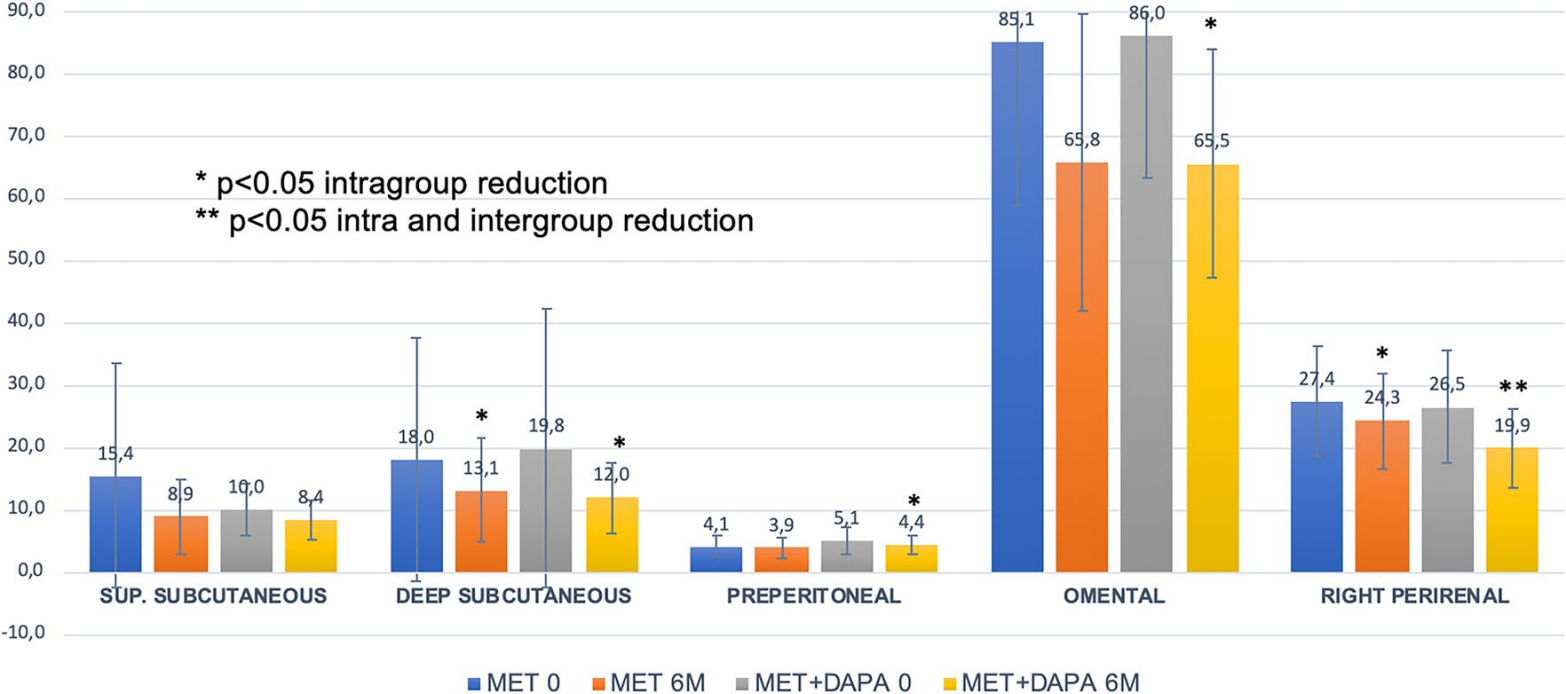
Abdominal fat layers reduction (mm).

Deep subcutaneous (*p* = 0.002); Preperitoneal (*p* = 0.03) and Right perirenal (*p* < 0.001) fat depots show significant reduction within the Met + Dapa group, whereas only deep subcutaneous (*p* = 0.03) and Right perirenal (*p* = 0.04) fat thickness reductions were seen with metformin-treated patients (Fig. 4).

**Figure 5:**
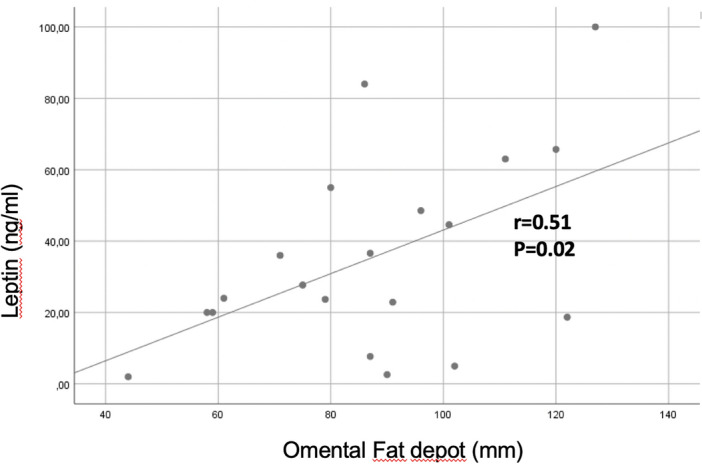
Omental fat correlations with leptin.

Omental fat loss correlated with leptin (r = 0.51 *p* = 0.02); CRP (r = 0.47 *p* = 0.002) and MAL (r = 0.41 *p* < 0.001) only in the Met + Dapa group (Fig. 5).

Right perirenal fat loss correlated with HOMA-IR (r = 0.74 *p* = 0.017) again only in the Met + Dapa group. We have’nt found significant correlations of leptin/Microalbuminuria when looking at perirenal fat. No correlation was found between different fat layers thickness and %weight loss or abdominal circumference reductions.

No changes in IMT were observed.

Significant HbA1c reduction at 6th month were seen in both cohorts (−0.9 ± 0.5% in group A −1.1 ± 0.7% in group B) but differences between cohorts did not reached statistical significance (*p* = 0.056). HOMA-IR was significantly reduced only in the combination group (−3.2 ± 1.6% in cohort A vs −6.7 ± 1.9% in B) (*p* = 0.012).

No significant differences were found between final and initial Total Cholesterol, LDL and Triglyceride values. No changes were seen for leptin or CRP or MAL at 6th month comparing the 2 groups.

No differences were seen adjusting by age, gender or initial weight.

### Side effects/safety

During this study, no serious adverse events (SAEs) were reported. A total of 12 patients shown AEs. 7 and 9 AEs were reported by patients treated with Metformin alone and Metformin + Dapagliflozin, respectively. Only 20.5% of the AEs were of moderate severity, 62.5% were AEs not related to study treatment. Gastrointestinal disorders (18.8%) were the most common described AEs. 13.8% of the patients reduced metformin dosage to 850 mg/day.

## Discussion

Compared with previous trials, our data shows mild to moderate weight loss with metformin alone (−5.6%) or added to dapaglifozin (−8.6%), mainly through abdominal fat reduction (−7.4 cm in abdominal perimeter at 6th months). Metformin insulin-resistance improvement and SLGT2i-induced glycosuria leading to net caloric output, added to a −500 kcal restriction diet, are possible mechanisms explaining weight loss. However, ultrasound (US) imaging techniques show significant reduction of abdominal fat layers, some of them (Omental −21% and Perirenal −27%), far beyond expected by weight-loss itself. US has allowed us to differentiate abdominal fat layers in detail and to describe pathological cut-off points beyond which metabolic and cardiovascular comorbidities may occur^11,20^. Preperitoneal and Omental fat depots, both related with insulin-resistance comorbidities, share the intraperitoneal space and are included in the classic concept of “visceral” obesity previously assessed by DEXA scan or CT imaging^33^. Perirenal fat depot, however, is located retroperitoneally and has recently emerged as a potential cardiovascular clinical marker^11,25,27^.

In previous studies, Preperitoneal fat has been linked with NAFLD^19^ or intima-media thickness^21^ in patients with high cardiovascular risk^20^. However, in our study, no correlations with cardiovascular biochemical markers were found with this particular layer. Only within the Metformin + Dapaglifozin group a Preperitoneal fat reduction was seen, without significance when comparing both treatment arms. PP fat responsiveness to Metformin/Dapaglifozin treatment is weak.

In both cohorts we observed a significant reduction of Omental fat depots after 6 months of treatment, independently of other confounding factors (age, gender or initial BMI). Intrabdominal fat reduction with metformin has already been proven in high fat diet animal models^35^ but this is the first evidence showing metformin efficacy in reducing omental fat depot. Omental fat is known to induce insulin-resistance through a deleterious adipokine secretion profile^14^ and is linked with cardiovascular morbidities^24^. Our study seems to support this hypothesis with some correlations between US-measured Omental fat and leptin or endothelial dysfunction markers (Ultrasensitive-CRP and microalbuminuria), especially in the intensified combined Metformin + Dapaglifozin treated group (although addition of dapaglifozin does not seem to induce greater omental fat loss to metformin alone). In cohort B, average Omental fat thickness reduction after 6 months of treatment was > 20 mm, allowing 21% of the patients to reach a “safety” level below the metabolic syndrome cut-off risk limits. Omental fat reduction should be considered as a clinical marker of insulin-sensitizing agents as Metformin or Dapaglifozin beneficial effect, in type 2 diabetes patients with obesity.

Perirenal fat also lowers significantly within both treatment arms, but it is the only abdominal fat depot showing significant differences when comparing both cohorts. The kidney involvement in metabolic and cardiovascular risk has become a breakthrough topic since the appearance of sodium-glucose cotransporter inhibitors SLGT2i as antidiabetic drugs^36^. They are also approved in the treatment of renal impairment^37^ and some mechanisms have been proposed to explain its capacity in preventing major cardiovascular events^6–9^. Major attention has been paid in measuring renal fat infiltration (MRI), particularly in sinus^38^. But little is known about the relationship between kidney’s surrounding Perirenal fat and cardiovascular risk. A cut-off limit (ROC curves) for Perirenal fat beyond which metabolic syndrome features occur (and presumably increased cardiovascular risk), have been published^11^. Its relationship with intima-media thickness and pericardial fat has also been pointed out, in children^39^. Perirenal fat is also considered a marker of subclinical atherosclerosis^40^ and modulates blood pressure control^41^ through a mechanistic effect. A bigger perirenal fat depot may difficult a congestive kidney to expand, leading to increased intra-medullar pressure and consequent hypertension (tamponade syndrome)^26^.

In our study, a significant reduction (24%) of Perirenal fat was found, particularly in the Metformin + Dapaglifozin group. 27% of Cohort’s B patients show a RK layer below 13 mm (cut-off for CV risk). Furthermore, we found a significant correlation between perirenal fat thickness and HOMA-IR, in both kidneys.

Could Dapaglifozin treatment prevent this increased intra-medullar pressure by reducing perirenal fat? Emerging biological markers (CA 125 or proBNP) have not been studied, and would help to answer that hypothesis^42^.

Unfortunately, we have not found correlations with other insulin-resistance biochemical markers such as leptin, microalbuminuria or C-reactive protein. We probably need to analyze cytokine expression directly, using tissue biopsy, to fully understand the differences between these different visceral layers and their correlations with insulin-resistance and cardiovascular markers.

We are aware that this study has some limitations. Although both treatment arms are very homogeneous and well balanced (age and gender), number of treated patients is small. The addition of a “diet-alone” arm would also have been interesting to highlight drug direct effects. We can consider these results as a proof-of-concept. Omental fat layer at baseline is particularly high (85 mm), clearly beyond the cut-off point for metabolic syndrome risk (> 54 mm), in both groups. Perirenal fat depot (27 mm) at baseline is also clearly beyond SM limits (13 mm), and microalbuminuria is in the upper normal limit at baseline (30 mg/24 h) in both treatment arms.

Long-term diabetic patients had been excluded and all were treatment-naïve, but their metabolic profile (ultrasound findings and biochemical cardiovascular markers) suggests a profound insulin-resistance at baseline. Whether this metabolic profile induces higher Omental/Perirenal fat loss, should be investigated. It is known that glycosuric effect is more pronounced at the beginning of iSLGT2 treatment, this may explain the greater weight loss observed in this short-term trial compared with previous published data. Would this effect in weight and ectopic fat reduction be maintained with longer treatment exposure? Although applied to both groups, in order to highlight the pharmacological effects, the initial −500 kcal dietary restriction, may also be a confounding factor, leading to major weight loss. The intensity of the dietary counseling follow-up, especially in newly diagnosed diabetic patients could be a confounding factor. Ad libitum diet would have reduced the met + dapa efficacy?.

## Conclusion

Treatment with Metformin and Metformin + Dapagliflozin in drug-naive type 2 diabetic patients are a safe and well-tolerated option. A decrease in the omental fat layer after 6 months of metformin treatment. Added Dapaglifozin increases effectivity in reducing perirenal fat. This study also shows specific correlations between omental and perirenal fat layers with multiple cardiovascular risk measurements.

US scan of abdominal fat layers can be a valuable tool to follow-up the efficacy of antidiabetic drugs with a cardiovascular perspective, and could also be used to explain the medical importance of visceral adiposity to our type 2 diabetic patients.

## Data Availability

All dataset records and statistical analysis performed by the CRO are fully available upon request at the principal investigator (gcuatrecasas@cpen.cat). Data sharing agreement with AstraZeneca would be asked.
